# Untangling the history of oroclines and mountain belts

**DOI:** 10.1093/nsr/nwab211

**Published:** 2021-11-26

**Authors:** Peter A Cawood

**Affiliations:** School of Earth, Atmosphere and Environment, Monash University, Australia

**Figure 1.**
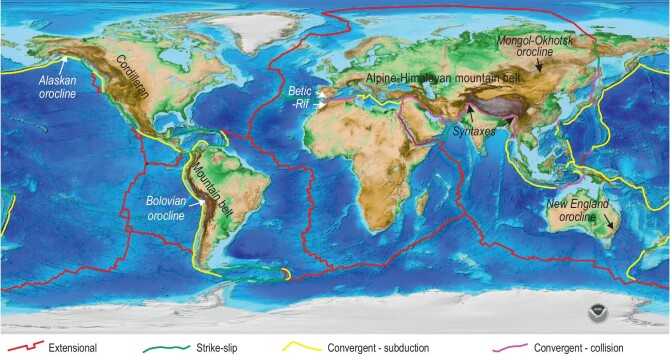
Present-day Earth global relief showing distribution of spreading, subduction, collisional and strike-slip plate boundaries. Modern oroclines (e.g. Alaskan, Betic-Rif, Bolivian and Himalayan syntaxes) lie along or close to active plate boundaries, whereas ancient oroclines (e.g. Mongol-Okhotsk, New England), which lay at plate boundaries at their time of formation, are now preserved in stabilized continental crust. Global relief model based on National Oceanic and Atmospheric Administration (NOAA) ETOPO1 1 Arc-Minute Global Relief Model [5].

Oroclines are map-view bends of the Earth's lithosphere formed by rotation of originally quasilinear linear rock units about a sub-vertical axis [1]. They can have amplitudes and wavelengths on the order of hundreds of kilometres and are a significant feature of many active and ancient mountain belts (Fig. 1, e.g. Alaska orocline, Bolivian orocline, Betic-Rif Belt, New England orocline and Himalayan syntaxes) [2,3]. Oroclines provide a record of the complex feedback between the evolving stress field forming a mountain belt and the various lithotectonic units of the belt (e.g. irregular continental margins). Mountain belts are amongst the major morphologic and tectonic features of the Earth, and are the sites for the formation and stabilization of the continents. They form through the opening and closing of ocean basins resulting in their positions either along the margins of, or within, a continent (Fig. 1, the accretionary Cordilleran mountain belt and the collisional Alpine-Himalayan mountain belt). Untangling their history and that of constituent elements such as oroclines is fundamental to understanding the history of our planet.

Wang *et al.* [4] provide a new and detailed analysis of the Mongol-Okhotsk orocline (Fig. 1), which lies in the eastern part of the Central Asian Orogenic Belt that extends through Mongolia, NE China and part of Russia. They document ages and compositional variations of Carboniferous to Jurassic magmatic activity (350–150 Ma) associated with a convergent plate margin that formed through closure of the Mongol-Okhotsk Ocean. Importantly, their data documents along- and cross-strike changes in magmatism. Wang *et al.* argue that the character of magmatic activity changes southward from a northwest–southeast orientated Andean-type continental margin into an east–west orientated intra-oceanic arc. The Andean margin experienced sustained trenchward migration driven by rollback of the subducting oceanic plate, relative to the southern section of the arc. Rotation of the arc system in response to ocean plate subduction took place around a centrally located vertical axis and resulted in the northwestern and southern segments moving into parallelism, leading to a west-to-east zipper-like final closure of the Mongol-Okhotsk Ocean.

The analysis of the Mongol-Okhotsk orocline undertaken by Wang *et al.* is made possible through their compilation of some 2660 new and previously published isotopic data, enabling them to map out the distribution of the magmatic activity. This approach reflects the impressive developments in mass spectrometry and microanalysis over the last quarter of a century, notably the growth of rapid laser induced coupled plasma (ICP) analysis. The generation and assembly of such large data sets have become increasingly important in untangling the history of mountain belts by providing information of high spatial and temporal fidelity that enables the testing of models for the generation and evolution of the Earth's outer rigid layer, the lithosphere.

Of course, new data and resultant models such as those presented by Wang *et al.* [4] for the Mongol-Okhotsk orocline, like all good science, are not the end of the story but rather they open up new avenues for future research. In particular, these expanding data sets, whether specific (e.g. geochemistry) or multifaceted (e.g. paleomagnetism, geochemistry, basin analysis and geophysics) allow us to better understand the 4D evolution of mountain belts as well as the Earth system as a whole. In the case of oroclines, future questions of research should include untangling the implications of 2D plan-view reconstructions for the 4D architecture of the lithosphere; i.e. how does the structure and composition of the whole lithosphere evolve within the orocline as well as the surrounding lithosphere?

***Conflict of interest statement*.** None declared.
